# Third stage of the kangaroo method: exclusive breastfeeding and growth of preterm and/or low birth weight newborns

**DOI:** 10.1590/1984-0462/2024/42/2023141

**Published:** 2024-04-29

**Authors:** Sandra Lima Ornelas, Rafael Paim Guimarães, Lorena Almeida Silva, Roberta Maia de Castro Romanelli, Maria Cândida Ferrarez Bouzada

**Affiliations:** a Fundação Hospitalar do Estado de Minas Gerais, Maternidade Odete Valadares – Belo Horizonte, MG, Brazil.; b Universidade Federal de Minas Gerais, Faculdade de Medicina – Belo Horizonte, MG, Brazil.

**Keywords:** Infant premature, Kangaroo-mother care method, Infant, newborn, Infant low-birth-weight, Breast Feeding, Growth, Pré-termo, Método canguru, Recém-nascido, Recém-nascido de baixo peso, Aleitamento Materno, Crescimento

## Abstract

**Objective:**

To evaluate the rates of exclusive breastfeeding (EBF) and growth of preterm and/or low birth weight newborns during the third stage of the Kangaroo Method (TSKM), at discharge.

**Methods:**

Retrospective study in a reference public maternity hospital between Jan/2014 and Dec/2017, including the preterm (less than 37 weeks) and/or low birth weight (less than 2500 g) newborn infants. Information was collected from medical records. Statistics analysis was done in SPSS software.

**Results:**

482 infants were included and followed up at the TSKM ambulatory. The average gestational age was 33 weeks (variation: 24-39 weeks) and birth weight, 1715g (variation: 455–2830 g). EBF occurred in 336 (70.1%) infants at hospital discharge, and in 291 (60.4%) at TSKM discharge. Each additional day of hospital stay increased the chance of infant formula (IF) use by 9.3% at hospital discharge and by 10.3% at TSKM discharge. Staying in the Kangaroo Neonatal Intermediate Care Unit (KNICU) favored EBF at hospital discharge and TSKM discharge (p<0.001). Not performing the kangaroo position increased the chance formula administration to the newborn infant at hospital discharge by 11%. Weight gain and head circumference growth were higher in infants using formula (p<0.001).

**Conclusions:**

The length of hospital stay and not performing the kangaroo position favored the use of infant formula at hospital and TSKM discharge. Staying in the KNICU favored exclusive breastfeeding at hospital and TSKM discharge. Weight gain and HC growth were higher in newborns receiving infant formula.

## RESUMO

**Objetivo:**

Avaliar as taxas de aleitamento materno exclusivo e o crescimento dos recém-nascidos (RN) pré-termo e/ou de baixo peso ao nascer à alta da terceira etapa do Método Canguru (TEMC).

**Métodos:**

Estudo retrospectivo realizado em maternidade pública de referência entre janeiro/2014 e dezembro/2017, que incluiu os RN pré-termo (<37 semanas) e/ou de baixo peso (<2.500 g). As informações foram coletadas de prontuários médicos e analisadas pelo programa *Statistical Package for the Social Sciences* (SPSS).

**Resultados:**

Foram seguidos 482 RN, com mediana da idade gestacional de 33 semanas (variação: 24–39 sem) e peso ao nascer 1.715 g (variação: 455–2.830 g). O aleitamento materno exclusivo (AME) ocorreu em 336 (70,1%) RN à alta hospitalar e em 291 (60,4%) à alta da TEMC. Cada dia a mais de permanência hospitalar aumentou a chance do uso de fórmula infantil em 9,3% à alta hospitalar e em 10,3% à alta da TEMC. Permanecer na Unidade de Cuidados Intermediários Canguru (UCINCA) favoreceu o AME à alta hospitalar e da TEMC (p<0,001). Não realizar a Posição Canguru aumentou em 11% a chance de o RN utilizar fórmula infantil à alta hospitalar. O ganho de peso e crescimento do perímetro cefálico foram maiores nos RN em uso de fórmula infantil (p<0,001).

**Conclusões:**

O tempo de permanência hospitalar e a não realização da Posição Canguru favoreceram o uso de fórmula infantil à alta hospitalar e da TEMC. Permanecer na UCINCA favoreceu o AME à alta hospitalar e da TEMC. O ganho de peso e crescimento do PC foram maiores nos RN em uso de fórmula infantil.

## INTRODUCTION

Prematurity and low weight at birth are the leading causes of infant mortality.^1^ In Brazil, the Kangaroo Method (KM) is a public health policy that proposes integral and humanized care for the newborn (NB), especially for preterm and low birth weight infants and their families. The KM has many advantages, including the promotion of breastfeeding and the emotional bond between mother-father-baby due to the Kangaroo Position (KP). That consists of keeping the NB at skin-to-skin contact, only wearing a diaper, upright, and close to the mother’s or father’s chest. The KM proposes three stages; the first and second occur during the hospital stay and the third after discharge when the baby is monitored in the outpatient clinic of the hospital where he was born and in Primary Care ambulatory.^2,3^ A multicenter Brazilian study showed a higher rate of exclusive breastfeeding (EBF) at hospital discharge in NB who underwent the KM, concluding that it is a safe alternative to conventional treatment and a good strategy for promoting breastfeeding.^4^ Randomized studies published by Cochrane showed that the KM is a protective factor for EBF at hospital discharge.^5^ In addition, an English study showed that there is an economic benefit in promoting breastfeeding and the Kangaroo Method in neonatal units.^6^

Many studies investigate various aspects of the Kangaroo Method, but those that address the TSKM were few found in the literature.^7-9^

Growth is an essential parameter among the various aspects to be evaluated in child health. In evaluating the growth of preterm and low birth weight infants, weight, length, and HC should be taken weekly or according to the child’s clinical conditions during hospitalization and after discharge in the outpatient clinic. For the follow-up of weight, length, and HC, some curves are published in the literature, with diverse methodologies, Intergrowth-21 being the most currently used. However, it includes a few extreme preterm infants.^10^

This study aimed to assess exclusive breastfeeding rates, weight gain, head circumference growth of preterm and/or low birth weight newborns at TSKM discharge, focusing on the hospital outpatient clinic.

## METHOD

A retrospective study was conducted in a public maternity hospital in Belo Horizonte/MG, concerning high obstetric and neonatal risk care from January 2014 to December 2017. A non-probabilistic sample of 482 preterm (less than 37 weeks) and/or low birth weight (less than 2500g) newborns attending the TSKM outpatient clinic were studied. Some newborns that were scheduled at the third stage outpatient clinic did not show up, probably for social reasons. They went directly to the primary care outpatient clinic closer to their homes (inside or outside the city) or were referred to specialized outpatient clinics. The number of absentees was not estimated in this study.

The TSKM discharge occurs when the NB reaches the weight of 2500g, the family is able to care for the baby at home and they have an appointment scheduled in the Primary Care ambulatory.

Information was collected from medical records and recorded in a database created in Excel. The independent variables were maternal age, delivery method, gender, gestational age, weight, head circumference, and classification (Fenton,2013 and Intergrowth 21)^10,11^ at birth, length of hospital stay, whether the Kangaroo Position was performed during hospitalization and the place where it was first started, type of diet and weight at hospital discharge. Regarding TSKM, the following data were collected: weight at admission and discharge, head circumference at discharge, number of consultations and type of diet. In the TSKM only the principal researcher obtained the weight and HC variables. The children were weighed entirely naked but wrapped in a swaddle or blanket, and their weight was deducted, according to the Kangaroo Method protocol. HC was measured with a standard, non-stretchable measuring tape, adjusted to the largest diameter of the head. The length measurement was excluded due to its more significant variability. The outcome considered the response variable as exclusive breastfeeding (EBF), mixed breastfeeding - breast milk and formula (MBF), or infant formula (IF). The curves adopted in the service for evaluating the growth of newborns about weight and HC at the time of the study were those of Fenton and Kim^11^ up to 40 weeks of corrected age, and after that age the WHO^12^ curves were used. Intergrowth-21 calculators performed reclassifications of weight and HC at birth and discharge from TSKM. SPSS was used for statistical analysis, and descriptive data analysis was performed, using measurements of central tendency (median), as all variables had a non-normal distribution, and percentage distribution of categorical variables, with distribution in the 25th and 75th percentiles. The chi-square test was used for comparison between proportions. Univariate and multivariate analysis were performed. Since the outcomes were categorical, the model applied was Multinomial Regression, considering p<0.20. Correlation analyses were carried out between explanatory variables that presented an association with the outcome; to this end, Spearman’s Correlation Test was used. Only the variables that maintained an independent association with the outcome (p<0.05) were kept in the final model. A 5% significance level was considered in all statistical tests. This project was approved by the Ethics Committee of the institution (FHEMIG-CAEE 3248618.1.0000), with waiver of the Informed Consent Form.

## RESULTS

Maternal, perinatal, and neonatal data related to the newborns attending the TSKM are summarized in Table 1. Regarding the type of diet, 336 (70.1%) NB were on exclusive breastfeeding (EBF) at hospital discharge and 291 (60.4%) at TSKM discharge (Table 1). 377 (82.5%) NB were placed in the Kangaroo Position (KP) during hospitalization. Not performing the KP increased the chance of the NB using FI at hospital discharge by 11% (Table 2). Staying in the Kangaroo Neonatal Intermediate Care Unit (KNICU) favored EBF at hospital discharge and TSKM discharge, p<0.001 (Table 3). Not remaining in the KNICU is related to 1.83 times (83%) the chance of the NB receiving mixed breastfeeding (MBF) compared to EBF at TSKM discharge (p=0.03, Table 4). Each additional day of hospitalization in the Conventional Neonatal Intermediate Care Unit (CNICU) increased the chance of IF use by 6.2%, and each additional day of hospital stay increased the use of IF at hospital discharge by 9.3%. In Table 4, it is observed that the only variable associated with IF to the discharge of TSKM when compared with EBF, was the length of hospital stay (p=0.00). Each additional day of hospital stay increases by 10.3% the chance of the infant having a formula diet at discharge from TSKM. The cesarean delivery increased the chance of mixed breastfeeding by 2.26 times compared to EBF at hospital discharge (p=0.005).

**Table 1:** Maternal, perinatal, and neonatal characteristics of 482 preterm and/or low birth weight newborns attending the TSKM, between January/2014 and December/2017.

Characteristics	n (%)
Mode of delivery (%)	
Vaginal	171 (35.5)
Caesarean	311 (64.5)
Gestational age – weeks (Median, Min, Max)	33 (24–39)
24–27 (extreme preterm)	18 (3.7)
28–30 (very preterm)	75 (15.6)
31–33 (pre-term)	190 (39.4)
34–36 (late preterm)	175 (36.3)
≥37 (full-term)	24 (5)
Weight at birth (Median, Min, Max)	1715 (455–2830)
≥ 2500 g	13 (2.7)
2499–1500 g	320 (66.4)
1499–1000 g	105 (21.8)
<1000 g	44 (9.1)
Weight at birth ≤1500 g	152 (31.5)
Weight at birth >1501 g	330 (68.5)
Head circumference at birth (Median, Min, Max)	29.5 (20–35)
Classification at birth (% SGA, AGA, LGA)	
Fenton	22.0/74.5/3.3
Intergrowth-21	27.6/69.7/2.7
Hospitalization time in days (Median, Min, Max)	18.0 (2–136)
Length of stay in NICU	5.0 (0–114)
Length of stay in CNICU	5.0 (0–76)
Length of stay in KNICU	5.0 (0–30)
KP during hospitalization	
Yes	377 (82.5)
No	80 (17.6)
Location in which KP performed for the first time	
NICU (n=321)	126 (27.5)
CNICU (n=316)	86 (18.8)
KNICU (n=285)	153 (33.4)
Type of diet (%): hospital discharge	TSKM discharge
EB 336 (70.1)	291 (60.1)
MB 124 (25.9)	167 (34.3)
IF 19 (4)	24 (5)

SGA: Small for Gestational Age; AGA: Appropriate for Gestational Age; LGA: Large for Gestational Age; NICU: Neonatal Intensive Care Unit; CNICU: Conventional Neonatal Intermediate Care Unit; KNICU: Kangaroo Neonatal Intermediate Care Unit; KP: Kangaroo Position, 25 (5.2% missing); EB: Exclusive Breastfeeding; MB: Mixed Breastfeeding; FI: Infant Formula; TSKM: Third Stage of the Kangaroo Method.

**Table 2:** Multinomial logistic regression of the association between preterm and/or low birth weight variables and the diet type, in relation to exclusive breastfeeding, at hospital discharge, of 482 newborns, between January/2014 and December/2017.

Variables associated with exclusive breastfeeding, at hospital discharge	Infant formula			Mixed breastfeeding		
	Coefficient	p-value	95%CI	Coefficient	p-value	95%CI
Days in CNICU	1.06	0.018	1.01–1.11	1.02	0.078	0.99–1.06
Weight gain (g)	1.003	0.002	1.00–1.00	1.00	<0.001	1.00–1.00
Caesarean delivery	1.01	0.979	0.26–3.88	2.26	0.005	1.27–3.97
Do not perform KP	11.00	0.005	2.09–57.84	1.53	0.267	0.072–3.24

CNICU: Conventional Neonatal Intermediate Care Unit, KP: Kangaroo Position.

**Table 3:** Association between preterm and/or low birth weight variables and diet type at hospital discharge and at discharge from the TSKM, of 482 newborns, between January/2014 and December/2017.

Variables	Hospital discharge				TSKM discharge			
	EB	MB	IF	p-value	EB	MB	IF	p-value
Days in CNICU Median (p25–p75)	3 (0–8)	8 (1.5–17)	27 (10–33)	<0.001	3 (0–8)	7 (0–15)	22.5 (5.5–30.75)	<0.001
Days in KNICU Median (p25–p75)	5 (0–8)	3 (0–10)	0 (0)	0.02	5 (0–8)	4.5 (0–9)	0 (0–9.5)	0.11
KP (%)								
Yes	265(82.8)	99 (85.3)	13(68.4)	0.19	220 (79.1)	139 (90.3)	17 (70.8)	0.04
No	55 (17.2)	17 (14.7)	6 (31.6)		58 (20.9)	15 (9.7	7(29.2
KNICU (%)								
Yes	234 (69.6)	69 (55.6)	3 (15.8)	<0.001	200 (68.7)	101 (60.8)	7 (29.2)	<0.001
No	102 (30.4)	55 (44.4)	16 (84.2)		91 (31.3)	65 (39.2)	17 (70.8)
Weight (g)								
Median (p25–p75)	1870 (1745–2005)	1960 (1822–2177)	2260 (2150–2810)	<0.001	1888 (1760–2010)	1922 (1803–2085)	2222 (2117–2470)	<0.001
Type delivery %								
Caesarean	203 (60.4)	95 (76.6)	10 (52.6)	0.03	169 (58.1)	129 (77.7)	13 (54.2)	<0.001
Vaginal	133 (39.6)	29 (23.4)	9 (47.4)		122 (41.9)	37 (22.3)	11 (45.8)
SGA (%)								
Fenton	78 (23.2)	25 (20.2)	4 (21.1)	0.8	69 (23.7)	33 (19.9)	5 (20.8)	0.713
Intergrowth	90 (26.8)	36 (29)	6 (31.6)	0.9	78 (26.8)	50 (30.1)	5 (20.8)	0.721

TSKM: Third Stage of the Kangaroo Method; EB: Exclusive Breastfeeding; MB: Mixed Breastfeeding; IF: Infant Formula; CNICU: Conventional Neonatal Intermediate Care Unit; KNICU: Kangaroo Neonatal Intermediate Care Unit; KP: Kangaroo Position; SGA: Small for Gestational Age.

**Table 4:** Multinomial logistic regression of the association between preterm and/or low birth weight variables and diet type, in relation to exclusive breastfeeding, at TSKM discharge, of 482 newborns, between January/2014 and December/2017.

Variables associated with exclusive breastfeeding. at TSKM discharge	Infant formula			Mixed breastfeeding		
	Coefficient	p-value	95%CI	Coefficient	p-value	95%CI
Weight gain (g)	1.00	0.271	0.99–1.00	1.00	0.005	1.00–1.00
Length of hospital stay (days)	1.10	<0.001	1.06–1.13	1.03	<0.001	1.02–1.05
Mother’s age	1.03	0.361	0.96–1.11	1.06	<0.001	1.02–1.09
Vaginal delivery	0.67	0.526	0.20–2.27	2.43	<0.001	1.46–4.06
Do not perform KP	4.26	0.081	0.83–21.77	0.40	0.029	0.18–0.91
Do not stay in KNICU	2.75	0.151	0.69–10.96	1.83	0.037	1.03–3.25

TSKM: Third Stage of the Kangaroo Method; KP: Kangaroo Position; KNICU: Kangaroo Neonatal Intermediate Care Unit.

There was a significant difference in weight gain and HC growth between types of diet at hospital and TSKM discharge, p<0.001 and p<0.001, respectively. Regarding NB weight at hospital discharge, the gain of each 100 g increased by 30% the chance of the NB using the FI at hospital discharge, p=0.002 (Table 2). The average percentages of HC gain from birth to discharge from TSKM, in patients using IF, was 39.4% (29.6-55.9). The weight gain (g/day) was greater in NB fed with MBF than in NB fed with EBF at TSKM discharge (p=0,005). The variable percentage of HC gain, despite having been significant in the univariate analysis, was not maintained in the multinomial logistic regression model as it had a high correlation with the variable of weight gain/day (Spearman correlation=0.642).

Regarding the NB classification at birth, whether small for gestational age (SGA), appropriate for gestational age (AGA), or large for gestational age (LGA), the percentage of newborns classified by the Fenton and Kim curve was respectively 22, 74.5 and 3.3%, while the percentage of those classified by the Intergrowth-21 was 27.6, 69.7 and 2.7%, showing that the classification by the Fenton curve underestimated the SGA (p<0,001).

It was found that between the length of hospital stay and the variables birth weight (r=0.80), gestational age (r=90.77), and days in the KNICU (r=0.75), there was collinearity. Therefore, these variables were removed for analysis of the type of diet at hospital discharge and at TSKM discharge, as well as the location of the first kangaroo position because it presented collinearity with the KNICU variable (p<0.01). All were removed from the linear regression analysis.

## DISCUSSION

In this study, it was found that staying in the Kangaroo Neonatal Intermediate Care Unit (KNICU) favored exclusive breastfeeding (EBF) at hospital and TSKM discharge. Souza et al.^13^ reported that the benefits of breastfeeding and the permanence of the low birth weight newborn baby in the KNICU during hospitalization acted positively and independently in EBF. Higher rates of EBF were found when comparing Kangaroo Care to Conventional Care at hospital discharge. Although most studies did not exactly report the rates of EBF at TSKM discharge, these were assessed at various intervals after hospital discharge. Comparing Kangaroo Care vs. Conventional Care, Almeida et al., in 2010, reported EBF at discharge of 82.6% vs. 0%, (p=0.00), at 40 weeks of 73.9% vs. 31.6% (p=0.01), at three months of 43.5 vs. 5.0% (p=0.005), and at six months of 22.7 vs. 5.9% (p=0.20).^14^ The same comparison was made by Lamy Filho et al., in 2008, who found 69.2% vs. 23.8%, (p=0.022).^4^ Penalva and Schwartzman, in 2006, showed EBF of 85.7% at discharge and 60.3% at six months.^15^ The drop in EBF rates at discharge from TSKM in our study was less than that observed by some authors at discharge from TSKM or later, such as Menezes et al. in 2014 (EBF of 56.2% at discharge and 14.4% at 6 months),^16^ Maia et al. in 2011 (EBF of 100% at discharge and 25% one week afterwards),^17^ and Cabral and Rodrigues in 2006 (EBF of 93% at discharge vs. 15.1% at third stage).^18^ The described studies highlighted that, although most of the newborns who received Kangaroo Care are discharged on EBF, at TSKM or later there is a significant reduction in EBF.

Alves et al., in 2020, reviewed 1328 national and international studies and selected 21 Brazilian studies that showed that KM has a positive influence on EBF and the establishment of the mother-child bond, however TSKM itself was not effective in maintaining breastfeeding. Therefore, it seems that KM favors the initiation of EBF, but is limited in maintaining breastfeeding in outpatient follow-up.^19^ As shown in our multivariate analysis, the length of hospital stay, especially if the newborn stays in the Neonatal Conventional Intermediate Care Unit (CNICU), increases the chance of IF use at discharge. Each additional day of hospital stay increases the chance of diet with IF at TSKM discharge. Inadequate management of breastfeeding during the hospital stay, especially in preterm and low birth weight infants may have favored the use of IF at hospital and TSKM discharge.

Similar to their infants, the mothers’ breasts are immature and require stimulation to increase and maintain milk production. Mothers whose babies are admitted to the Neonatal Intensive Care Unit (NICU) or to the CNICU should be encouraged to start expressing their own milk between 6 and 12 hours after birth and to maintain milking 6 to 12 times a day.^20^ This intervention stimulates milk production and increases the likelihood that the preterm baby will receive his or her own mother’s milk. During outpatient follow-up, the maintenance of EBF is a challenge for providers in TSKM. When babies are discharged from the maternity hospital and remain at home with their families, cultural and social factors seem to have more impact on breastfeeding, and careful counseling is required. Cabral and Groleau^21^ highlight the importance of home visits for encouragement, guidance, and maintenance of breastfeeding, with the involvement not only of the mother, but of the entire family and community.

It is important to emphasize that most of the newborns in the study were placed in the Kangaroo Position with their mother and/or father during hospitalization and, in one third of them, the procedure was performed for the first time in the KNICU, i.e., in the second stage of KM, showing that the initiation of skin-to-skin was late. Jayaraman et al.^22^ reported that the early Kangaroo Position, in relation to the late procedure, improves feeding with expressed breast milk (86 vs. 45%, p<0.001) or direct breastfeeding (49 vs. 30%, p=0.021) during hospitalization and one month after discharge (73 vs. 36%, p<0.001).

On the other hand, the data from the present study shows that the non-permanence in the KNICU is related to MBF in relation to EBF, at discharge from TSKM. Not carrying out the Kangaroo Position at any time during hospitalization increases the chance of IF administration at hospital discharge by 11%. Tully et al.^23^ found that skin-to-skin alone did not appear to have a positive effect on breastfeeding during hospitalization, on the duration of breastfeeding or EBF after discharge in preterm neonates in the absence of other support mechanisms; however, stimulating breastfeeding during hospital stay was positively associated with its maintenance after discharge (p<0.001). According to Goudard et al.,^24^ the duration of skin-to-skin contact greater than 149.6 min/day showed a strong association with EBF at hospital discharge (p<0.001).

Cesarean delivery increased the chance of mixed breastfeeding at hospital discharge. It was seen that 377 (64.5%) newborns were delivered by cesarean, a fact that may be associated with the birth of high risk babies who required prolonged hospitalization.

Newborns fed by IF had greater weight gain and HC growth at hospital and at TSKM discharge than those fed by EBF or MBF. These data agree with the literature, which show that NB gain weight more rapidly when they are formula-fed. According to Quigley et al.,^25^ formula-fed infants had higher in-hospital rates of weight gain: 1.93 to 3.08 g/kg/day (MD 2.51, 95% CI), and head growth: 0.47 to 1.23 cm/week (MD 0.85) but increased risk of necrotizing enterocolitis (RR 1.87, 95% CI, 1.23 to 2.85; RD 0.03, 95%CI, 0.01 to 0.06). According to Lucas et al.,^26^ formula-fed babies have greater weight gain, but those fed exclusively with mixed breast milk had better intelligence quotients. Freitas and Camargo^27^ studied weight gain of NB in the second stage of KM, and 54.6% were below the 5th percentile at discharge; the relative speed of weight gain of NB with EBF or mixed feeding was 11.9 g/kg/day and 14.5 g/kg/day, respectively. Menezes et al.^16^ reported that 68.4% (95%CI 56.7–78.6) of the NB assisted by the Kangaroo Method were, at six months of chronological age, between the 15th and 85th percentiles of the WHO weight curve, on average, weighing 5,954±971g, with 14.4% in EBF (95%CI 8.1–23). Gathwala et al,^28^ in a randomized controlled trial of 100 late preterm, showed the following in the kangaroo and control groups: weight gain of 21.92±1.44g/day vs. 18.61+1.28g/day (p<0.05), and head circumference growth of 0.59±0.04cm/week vs. 0.47+0.03cm/week (p<0.05) at 3 months of corrected age. According to Boundy et al.,^29^ newborns cared by the Kangaroo Method had better HC growth (0.19 cm/week greater than the control group, p=0.04). The mentioned studies provided important parameters on weight gain and HC growth of NB assisted by the KM, but they do not specifically address growth during or at TSKM discharge. The HC measure is an important parameter to evaluate the NB growth.^30^ In this study, the variable percentage of HC gain had a high correlation with the variable of weight gain/day; therefore, only the variable weight gain/day was maintained in the final regression model.

Regarding the NB classification at birth, the percentage of SGA babies classified by Intergrowth-21th was greater than those SGA assessed by Fenton and Kim curves. From 2017 on, Intergrowth-21st curves were the standard at Maternidade Odete Valadares, where this study was performed, as it has a more appropriate methodology for evaluating the growth of preterm newborn. For this reason, and because it is currently used in most Brazilian services, reclassification at birth and calculation of the measurement percentiles using the Intergrowth-21 calculators was carried out.

On average, few consultations were performed for each NB during TSKM. The number of patients absent from consultations or the reason for their absence was not recorded, although an active search for absentees was conducted. Other studies have shown that a reduction in the number of outpatient visits was a risk factor for early weaning. Moreover, it was pointed out that the difficulty of access related to the distance from home is a barrier to TSKM.^7,10^ This fact was a limitation of this study to comply with the main norm of the TSKM discharge, which is the weight of 2500 g. For the families that live outside of Belo Horizonte, many times with few financial resources, we opted for TSKM discharge before the NB reaches 2500 g, provided there is a Primary Care ambulatory in their cities.

Data regarding hospital stay of NB were obtained from medical records. After discharge, only one neonatologist was responsible for all consultations and performed data collection at the TSKM. The fact that this was a retrospective study made it difficult to obtain information from a standardized template.

This study concluded that staying in the Kangaroo Neonatal Intermediate Care Unit (KNICU) during hospitalization favored EBF at hospital and TSKM discharge. Not performing the Kangaroo Position during the hospitalization favored IF use at discharge. The newborns fed by IF had greater weight gain and head growth than those fed by EBF or mixed feeding at hospital discharge. The clinics outcomes were not evaluated. The TSKM mirrors the approach to the NB and its family during hospitalization, showing its importance especially for preterm and/or low birth weight NB follow-up.

## Data Availability

The database that originated the article is available with the corresponding author.
